# TCMP-300: A Comprehensive Traditional Chinese Medicinal Plant Dataset for Plant Recognition

**DOI:** 10.1038/s41597-025-05522-7

**Published:** 2025-07-09

**Authors:** Yanling Zhang, Wanhui Sun, Chuanguang Yang, Libo Huang, Zhulin An, Weilun Feng, Wenjing Tang, Yongjun Xu

**Affiliations:** 1 School of pharmacy, Xinyang Agriculture and Forestry University, Xinyang, 464000 China; 2 Institute of Computing Technology, Chinese Academy of Sciences, Beijing, 100190 China; 3 University of Chinese Academy of Sciences, Beijing, 100190 China

**Keywords:** Plant sciences, Biological techniques

## Abstract

Traditional Chinese Medicinal Plants (TCMPs) are often used to prevent and treat diseases for the human body. Since various medicinal plants have different therapeutic effects, plant recognition has become an important topic. Traditional identification of medicinal plants mainly relies on human experts, which does not meet the increased requirements in clinical practice. Artificial Intelligence (AI) research for plant recognition faces challenges due to the lack of a comprehensive medicinal plant dataset. Therefore, we present a TCMP dataset that includes 52,089 images in 300 categories. Compared to the existing medicinal plant datasets, our dataset has more categories and fine-grained plant parts to facilitate comprehensive plant recognition. The plant images were collected through the Bing search engine and cleaned by a pretrained vision foundation model with human verification. We conduct technical validation by training several state-of-the-art image classification models with advanced data augmentation on the dataset, and achieve 89.64% accuracy. Our dataset promotes the development and validation of advanced AI models for robust and accurate plant recognition.

## Background & Summary

Medicinal plants refer to plants that prevent and treat diseases to maintain health for the human body. Medicinal plants are not only the source of a large number of Western medicinal compounds but also the main source of Complementary and Alternative Medicine (CAM). China is rich in medicinal plant resources. According to statistics^1^, there are 11,146 species belonging to 383 families and 2313 genera of medicinal plants in China. In recent years, medicinal plants have played an increasingly important role in the prevention and treatment of various diseases^2–4^. However, different medicinal plants have distinct therapeutic effects. In clinical practice, it is necessary to use them according to the specific condition to mitigate adverse reactions and avoid toxicity. Therefore, accurately identifying medicinal plants to avoid misplanting, miscollection, misuse, and fraudulent substitution is the most fundamental and crucial step in ensuring the quality and efficacy of medicines.

As a part of systematics, taxonomy is closely related to phylogenetic studies and the research of evolutionary processes within systematics. Its tasks include using the findings of systematics to name and classify organisms under reasonable principles, providing an organizational framework that facilitates the storage and retrieval of biodiversity information, and offering guidance for identification work^5^. Plant taxonomy is a long-established discipline that has continuously developed and improved through the practice of human recognition and utilization of plants. Guided by Darwin’s theory of evolution^6^, plant taxonomists from various countries have proposed insights into plant classification systems by studying the origin and development of the plant kingdom. Among the most influential systems are those of Engler, Hutchinson, Takhtajan, and Cronquist. The Engler system and the Hutchinson system, in particular, represent the “Pseudanthium” and “Euanthium” schools, respectively, with flower characteristics being the primary distinguishing features^7^. The classification of medicinal plants adopts the principles and methods of plant taxonomy. Mastering characteristics such as plant morphology and microscopic structure ensures the accurate identification of plants with medicinal value^8^. This guarantees the authenticity of medicinal plants and herbal medicines, facilitating better research, rational development, and utilization.

Traditional identification of medicinal plants mainly relies on professionals according to the corresponding classification system. According to the written description of the classification system, the identification of medicinal plants is carried out by sight, hand touch, mouth taste, and nose^9^. This method mainly depends on the professional level and experience of the operator. Therefore, different operators have certain subjectivity in identifying medicinal plants, and their accuracy rates vary. With the development of human society, the demand for medicinal plant resources is increasing. Traditional identification methods cannot adapt to the rich diversity of plant taxa. At present, the classification of medicinal plants can be conducted from different perspectives, including pharmacognosy, pollen, leaf epidermis and seed coat, cytology, and molecular identification^10^.

Particularly, plant image recognition technology based on deep learning^11,12^ is a more intelligent and reliable auxiliary technology that can improve the accuracy and efficiency of plant identification. Due to the higher difficulty in collection and annotation, there are relatively fewer related works. Besides, Traditional Chinese Medicine (TCM) is more prevalent in Southeast Asia, and research on Traditional Chinese Medicinal Plant (TCMP) datasets is also distributed in the Southeast Asian region. In 2021, Tung and Vinh^13^ proposed a large, public, and multi-class dataset of medicinal plant images called VNPlant-200. The dataset consists of a total of 20,000 images of 200 different labeled Vietnamese medicinal plants. They also reported the preliminary classification results by using two local image descriptors. Huang *et al*.^14^ built a Chinese medicinal blossoms dataset, which consists of twelve blossoms used in TCM. The authors employed various data augmentation methods to increase the number of samples and provided the image classification results using AlexNet^15^ and InceptionV3^16^. Abdollahi^17^ proposed using MobileNet^18^ to identify various medical plants in Ardabil, Iran. The dataset used to train and evaluate includes 30 different classes of medicinal plants, totaling 3000 images. DIMPSAR^19^ is a dataset for the analysis and recognition of Indian medicinal plant species. The dataset consists of two parts: leaf and plant. The former includes 80 classes, while the latter contains 40 classes. MED-117^20^ is an image dataset of common medicinal plant leaves from Assam, India. Beyond classification, the MED-117 dataset also includes segmented leaf frames for leaf segmentation using U-Net^21^. Ref. ^22^ reported a collection of leaf images from 10 common medicinal plants in Bangladesh. This paper also used several common Convolutional Neural Networks (CNNs) to classify this dataset. In addition to medicinal plant datasets, researchers have also collected an image dataset of TCM fruits^23^. This dataset includes 20 common types of TCM fruits. This paper also utilizes CNNs for classification. We summarized the details of the above datasets in Table 1.

**Table 1:** Detail information about related datasets.

Name	Time	Country/Region	Part	Class Nums	Image Nums (Augmented Nums)	Resource
VNPlant-200^13^	2021	Vietnam	Plant	200	20000	Self-built
Chinese Medicinal Blossom^14^	2021	Taiwan	Blossom	12	1716 (12538)^*^	Google
Medicinal Plants in Ardabil^17^	2022	Iran	Plant	30	3000	Unreported
MED 117^20^	2023	India	Leaf	117	77500	Self-built
DIMPSAR^19^	2023	India	Leaf	80	6900	Self-built
			Plant	40	5900	
Bangladeshi Medicinal Plant^22^	2023	Bangladesh	Plant and leaf	10	5000	Self-built
NB-TCM-CHM^23^	2024	China	Fruits	20	3384+400	Google+Self-collected
Our	2025	China	All	300	52089	Bing

* After data augmentation.

However, although some works of medicinal plant datasets have been introduced, there are still many challenges in this field:

1. **Limited diversity of species**. Existing medicinal plant datasets capture a limited number of plant species and parts, restricting their application in clinical practice.
2. **Lacking systematic data collection and cleaning framework**. The data collection and cleaning process for existing datasets often highly relies on manual curation without automation tools.
3. **Inadequate validation framework**. Previous papers do not conduct technical validation using state-of-the-art network architectures and advanced data augmentation methods. This hampers the accurate assessment of datasets and achieves suboptimal performance.

**Fig. 1:**
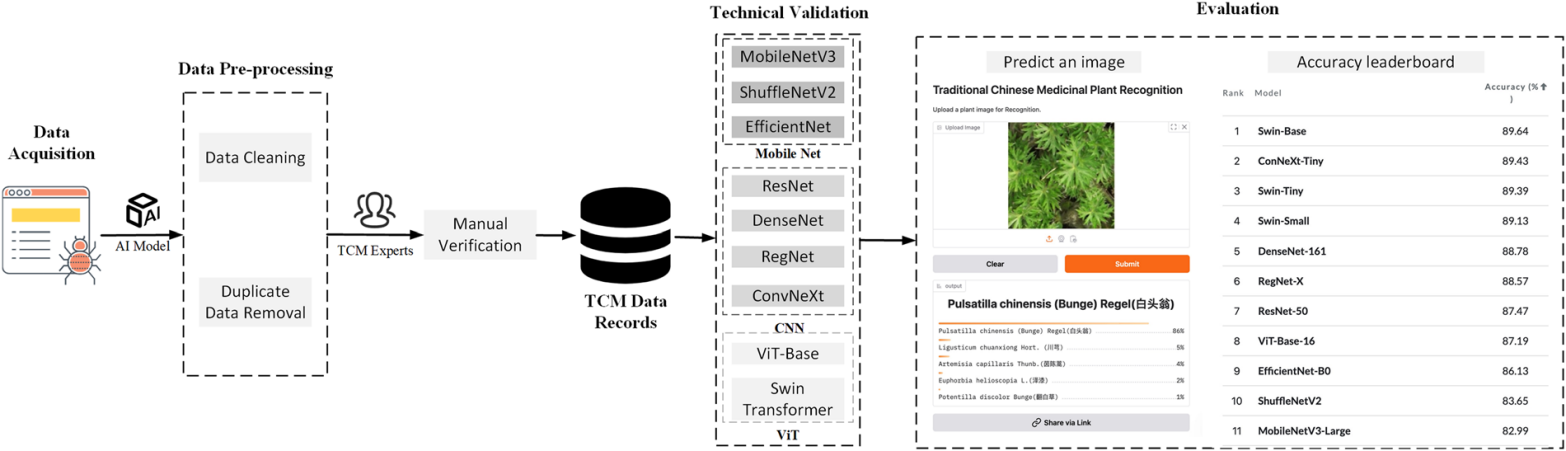
Overview of the data processing workflow and evaluation for our TCMP-300 dataset.

To address these challenges, our TCMP-300^24^ dataset stands out with its comprehensive design and structured methodology. The overview of the overall data processing workflow and evaluation is illustrated in Fig. 1. By leveraging an automated web crawler, we have ensured a diverse representation of species and plant parts, capturing images from various angles and contexts. This not only enhances the dataset’s ability to reflect the true diversity of medicinal plants but also mitigates the limitations posed by manual curation. Furthermore, our systematic data-cleaning process ensures that only high-quality and relevant images are included, providing a strong foundation for subsequent research and model training. The final dataset is verified by human TCM experts. We conduct comprehensive technical validation using three popular image-classification network families, including mobile network, CNN, and Vision Transformer (ViT). Additionally, we propose an advanced data augmentation technique, HybridMix, during the training process, which not only enriches the dataset but also enhances the robustness of models. The largest Swin-Base model^25^ achieves 89.64% accuracy on our TCMP-300^24^ dataset. Our dataset, combined with released superior models, set a new standard for future medicinal plant recognition research, making it easier for researchers to reproduce results and build upon our findings.

Among the selected medicinal plants, the most species belong to the following families: Asteraceae (27 species), Lamiaceae (18 species), Liliaceae (14 species), Ranunculaceae and Fabaceae (11 species each), Apiaceae (10 species), Brassicaceae and Solanaceae (8 species each), Rosaceae, Papaveraceae, Scrophulariaceae, Araliaceae, and Campanulaceae (6 species each), while other families contain fewer than 5 species. The selected medicinal plants predominantly contain bioactive compounds such as flavonoids, alkaloids, volatile oils, coumarins, sterols, and tannins. They exhibit clinical effects such as (1) clearing heat and detoxifying; (2) antibacterial and anti-inflammatory; (3) relieving cough and resolving phlegm; (4) reducing swelling and dissipating nodules; (5) promoting blood circulation and resolving stasis. Among them, certain folk medicinal herbs such as bittersweet, black nightshade, and rorippa have not yet been fully explored and utilized. These plants hold significant potential as valuable resources for developing new traditional Chinese medicines in clinical practice.

In summary, the comprehensive and meticulously curated TCMP-300^24^ dataset represents a significant advancement in the field of medicinal plant research. By facilitating the classification and identification of diverse plant species, this dataset not only enhances our understanding of the medicinal properties of these plants but also accelerates the discovery of new therapeutic agents. For example, the development of artemisinin, a Nobel Prize-winning treatment for malaria, was the result of extensive manual research to identify effective compounds. With a well-structured dataset, researchers could potentially streamline the process of discovering new drugs from nature, significantly improving efficiency in the search for novel therapies. Moreover, this dataset aligns with the United Nations Sustainable Development Goal 3: “Ensure healthy lives and promote well-being for all at all ages.” By providing a robust resource for the classification and study of medicinal plants, it can support initiatives aimed at improving healthcare and promoting the use of traditional medicine. Ultimately, the development of such datasets not only contributes to scientific knowledge but also has the potential to foster sustainable practices in healthcare and biodiversity conservation, paving the way for a healthier future for all.

## Methods

### Image crawling

**Fig. 2:**
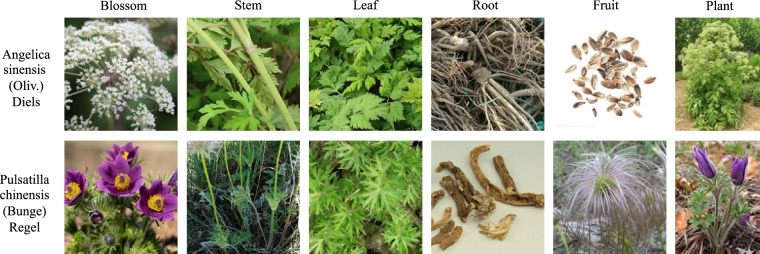
Examples in TCMP-300 dataset.

The dataset images were collected through the Bing search engine using an automated web crawler that queried with standardized scientific names (following the binomial nomenclature system: Genus species + authority, *e.g*., Angelica sinensis (Oliv.) Diels and Pulsatilla chinensis (Bunge) Regel) of medicinal plants. After initial automated retrieval, all images underwent rigorous manual and taxonomic validation to ensure botanical fidelity. The final collection includes high-quality images depicting key morphological features of each plant species, such as blossoms, stems, leaves, roots, fruits, and whole-plant profiles, with representative examples shown in Fig. 2.

### Data cleaning

**Fig. 3:**
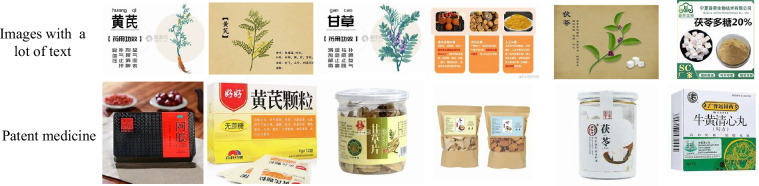
Examples of dirty sample.

**Fig. 4:**
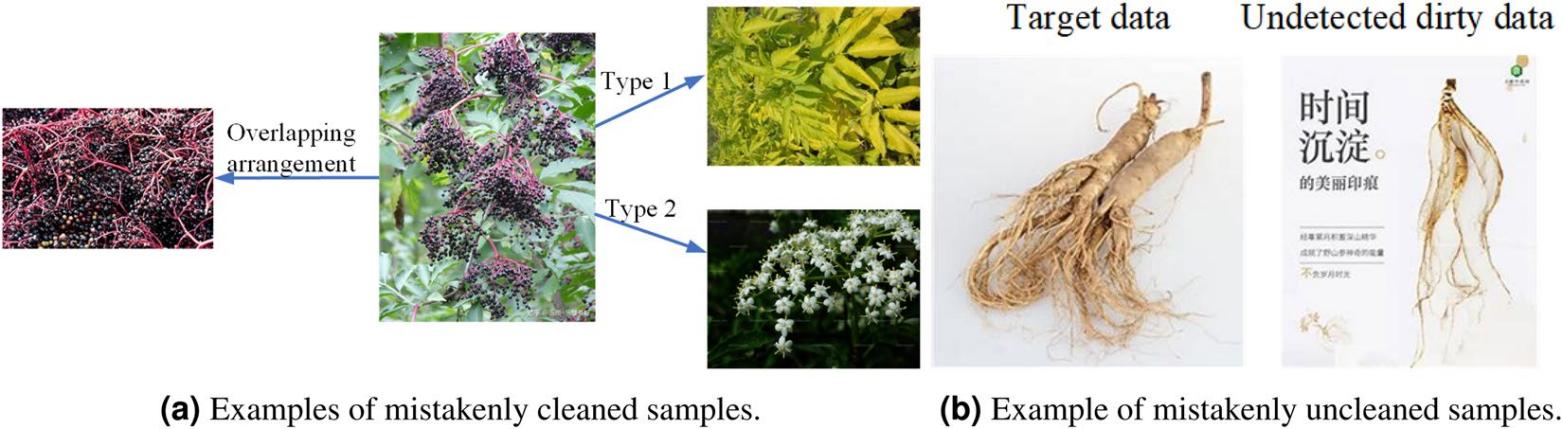
Examples of misclassification or overlooked samples.

**Fig. 5:**
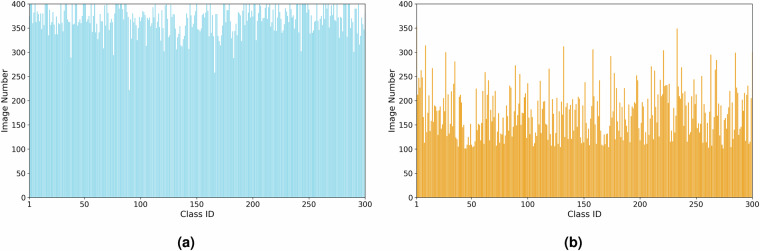
Comparison of the number of images before and after data cleaning.

The data-cleaning process began with the development of textual prompts based on medicinal plant labels, followed by the application of CLIP (Contrastive Language-Image Pre-Training)^26^, a robust vision foundation model, to classify the crawled images. Guided by TCM experts, precise classification thresholds were established for each category, enabling the identification and removal of low-quality samples. Figure 3 illustrates examples of dirty data, such as images containing textual content or pharmaceutical products, which were prevalent in the initial dataset. After the first cleaning phase, we eliminated 52.73% noise images in the initial dataset. Figure 5 compares the number of images per class before and after cleaning, showing a significant reduction from 222~400 to 101~354 images per class, highlighting the poor quality of the original data. To further refine the dataset, TCM experts conducted manual verification, addressing misclassified or overlooked samples. Figure 4 demonstrates two types of errors: mistakenly cleaned samples (e.g., rare variants of “Sambucus williamsii” misclassified due to atypical appearances or obscured features) and mistakenly uncleaned samples (e.g., images with ambiguous visual features or subtle text overlays that evaded automated detection). This comprehensive approach ensured a high-quality dataset for subsequent analysis. Notice that few images in the TCMP-300^24^ dataset may also inevitably contain visible watermarks or embedded annotations (e.g., numbers). According to the observation from Northcutt *et al*.^27^, the noisy data may be a common phenomenon in computer vision machine learning datasets, such as MNIST^28^, CIFAR-10^29^, CIFAR-100^29^, Caltech-256^30^, and ImageNet^31^. This further demonstrates that the noisy data is not a problem specific of our dataset but a reality of the computer vision field.

## Data Records

### Folder structure and recording format

The TCMP-300^24^ dataset, publicly available at 10.6084/m9.figshare.29432726, comprises 52,089 images across 300 TCMP categories. The user could download “tcmp-300-release.tar.gz” and “tcmp-info-20250329.csv”, where the former contains TCMP images and the latter describes class information. The user should run the script “tar -zxvf tcmp-300-release.tar.gz” to extract image files. The dataset is organized into category-specific subfolders following the naming convention “[Class ID].[English Name of TCMP]” (*e.g*., “001.Veronica persica Poir.”), with each subfolder containing all corresponding medicinal plant images. All images are provided in widely compatible formats (PNG, JPG, JPEG, and WebP), ensuring seamless integration with modern AI frameworks such as PyTorch^32^.

### Data Splitting

Because our dataset exhibits a long tail distribution, to maintain this data characteristic, we divide the dataset based on classes as the basic unit. To split the whole dataset into train and validation subsets, we first define a threshold *τ*. For class *C*_*i*_ which contains ∣*C*_*i*_∣ images, *i* ∈ [1, 2, ⋯ , *n*], where *n* stands for the total class number. We can calculate the image number of its corresponding train subset and validation subset by Equ. (1):

1 $${N}_{train}^{{C}_{i}}=\lfloor \tau \times | {C}_{i}| \rfloor ,\,{N}_{validation}^{{C}_{i}}=| {C}_{i}| -{N}_{train},$$

where ${N}_{train}^{{C}_{i}}$ and ${N}_{validation}^{{C}_{i}}$ are the number of images from the training subset and validation subset for class *C*_*i*_, respectively. ⌊⋅⌋ denotes round down operation. In our practice, we set *τ* = 0.7.

### Properties

To make our dataset suitable for a wide range of identification tasks in practice, the TCMP-300^24^ dataset has three valuable properties:

- **Comprehensiveness**. Our dataset covers the maximum number of TCMP categories compared to previous datasets. It encompasses six major parts of TCMP: blossom, stem, leaf, root, fruit, and the whole plant. This extensive categorization ensures a complete representation of various TCMP components.
- **Diversity**. To augment the dataset’s diversity, images were scraped from the internet encompassing various perspectives, both indoor and outdoor settings, a spectrum of lighting conditions, and complex backgrounds. This mirrors the multifaceted contexts where medicinal plants are typically found. Our dataset merges images captured from diverse environments to ensure the generalization of TCMP recognition.
- **Long-tailed distribution**. The distribution of instances across various categories emulates the real-world prevalence of TCM. The image number of frequently utilized medicinal plants substantially surpasses that of their rare counterparts, as illustrated in Fig. 5. This long-tailed distribution requires the model to achieve good performance on the overall categories, even if the sample numbers in some categories are small.

## Technical Validation

### Model architectures

We adopt popular image classification models, including ResNet^33^, DenseNet^34^, RegNet^35^, MobileNetV3^36^, ShuffleNetV2^37^, EfficientNet^38^, ConvNeXt^39^, ViT^40^, and Swin Transformer^25^, to evaluate the dataset. The first seven models are CNN backbones, where MobileNet, ShuffleNet, and EfficientNet are lightweight networks for edge devices. The last two models are visual Transformer networks.

### Classification framework

**Fig. 6:**
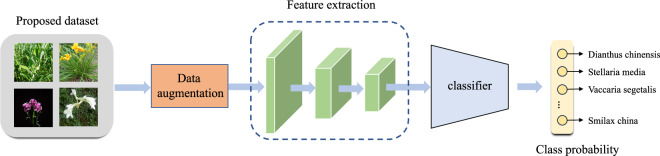
Pipeline of the TCMP image classification framework.

Figure 6 shows the pipeline of the TCMP image classification framework. The input is an image ***x*** with RGB channels. We first adopt a data augmentation module *τ*(⋅) to preprocess the input image and produce an augmented image $\widetilde{{\boldsymbol{x}}}$, *i.e*., $\widetilde{{\boldsymbol{x}}}=\tau ({\boldsymbol{x}})$. A general image classification network *f* is applied to classify the augmented image $\widetilde{{\boldsymbol{x}}}$, *e.g*., ResNet. The classification network *f* often includes a feature extractor *ϕ* for feature learning and a linear classifier *ξ* to output class probability distribution ${\boldsymbol{p}}=f(\widetilde{{\boldsymbol{x}}})=\xi (\phi (\widetilde{{\boldsymbol{x}}}))\in {{\mathbb{R}}}^{C}$, where *C* is the number of classes. The feature extractor *ϕ* includes *L* stages ${\{{\phi }_{l}\}}_{l=1}^{L}$ to process image features. Each stage *ϕ*_*l*_ downsamples the input image to refine hierarchical information and generate semantic features. We adopt the traditional cross-entropy loss to the image classification network. Given the input image ***x*** and its ground truth *y*, the cross-entropy loss is formulated as Equ (2).

2 $$L=-\mathop{\sum }\limits_{c=1}^{C}{\tau }_{c,y}\log \,{{\boldsymbol{p}}}_{c}.$$

Here, *τ*_*c*,*y*_ is an indicator function. If *c* = *y*, *τ*_*c*,*y*_ = 1, else wise *τ*_*c*,*y*_ = 0.

### Data augmentation

Following the ImageNet classification, we utilize random cropping and flipping as the standard data augmentation^41^. The input image is first randomly cropped, ranging from 0.08 to 1.0 of the original image size. The aspect ratio is ranged from 3/4 to 4/3 of the original image aspect ratio. The cropped image is resized to 224 × 224 and horizontally flipped using a probability of 0.5. Finally, it is normalized by the mean and standard deviation over RGB channels.

**Fig. 7:**
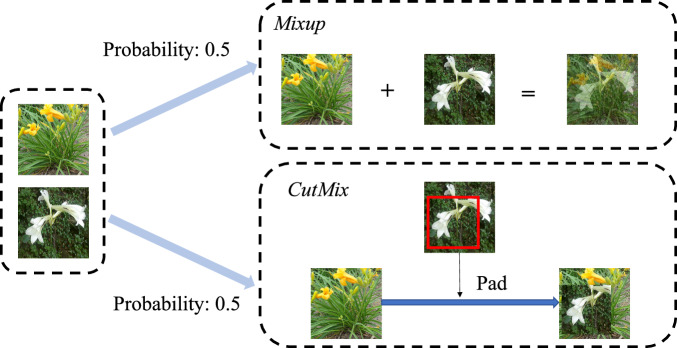
Overview of HybridMix framework.

We further propose HybridMix, an image mixture technique, as the extra data augmentation to improve the model accuracy. As shown in Fig. 7, given an input image, HybridMix chooses Mixup or CutMix with a probability of 0.5 to perform global or local image mixture.

1. **Global Image Mixture: Mixup**^42^. Given two different images ***x***_*i*_ and ***x***_*j*_ from the mini-batch, Mixup conducts global pixel mixture using a balancing factor *λ* to produce a new mixed image ${\widetilde{{\boldsymbol{x}}}}_{ij}$, where *λ* ∈ [0, 1]. The label of the mixed image is also linearly interpolated between *y*_*i*_ and *y*_*j*_ with the same balancing factor *λ* to generate mixed label ${\widetilde{y}}_{ij}$. The formulations of ${\widetilde{{\boldsymbol{x}}}}_{ij}$ and ${\widetilde{y}}_{ij}$ are shown as Equ (3).
  3 $${\widetilde{{\boldsymbol{x}}}}_{ij}=\lambda \ast {{\boldsymbol{x}}}_{i}+(1-\lambda )\ast {{\boldsymbol{x}}}_{j},\,{\widetilde{y}}_{ij}=\lambda \ast {y}_{i}+(1-\lambda )\ast {y}_{j}$$
2. **Local Image Mixture: CutMix**^43^. The main idea of CutMix is to crop a patch from ***x***_*j*_ and pad ***x***_*i*_ with the cropped patch. We represent the bounding box coordinates of the cropped patch on ***x***_*j*_ as **B** = (*r*_*x*_, *r*_*y*_, *r*_*w*_, *r*_*h*_), where ${r}_{w}=W\sqrt{\lambda }$ and ${r}_{h}=H\sqrt{\lambda }$, *λ* ∈ [0, 1], *W* and *H* are the width and height of the image, respectively. *r*_*x*_ and *r*_*y*_ are uniformly sampled according to *r*_*x*_ ~ *U*(0, *W*), *r*_*y*_ ~ *U*(0, *H*). The CutMix image is generated by removing the patch **B** on ***x***_*i*_ and filling the corresponding patch cropped from ***x***_*j*_. In practice, a binary mask **M** = {0, 1}^*W*×*H*^ fills 0 according to the bounding box **B** otherwise 1. The output CutMix image and interpolated label can be expressed as:
  4 $$\widehat{{\boldsymbol{x}}}={\bf{M}}\odot {{\boldsymbol{x}}}_{i}+({\boldsymbol{1}}-{\bf{M}})\odot {{\boldsymbol{x}}}_{j},\,{\widetilde{y}}_{ij}=\lambda \ast {y}_{i}+(1-\lambda )\ast {y}_{j}.$$
  Here,  ⊙ denotes the element-wise multiplication, ***1*** = {1}^*W*×*H*^ is the binary mask filled with 1.

### Training setup

All models are trained by a stochastic gradient descent (SGD) optimizer with a momentum of 0.9, a weight decay of 1 × 10^−4^, and a batch size of 128. We utilize a cosine learning rate strategy, the learning rate of which starts at 0.1 and gradually decreases to 0 within the total 300 epochs.

### Evaluation metrics

We utilize Accuracy (Acc), F1 score, Balanced Accuracy (B-Acc), and Balanced F1 score (B-F1) to measure the performance of TCMP recognition. The predicted results are often constructed by True Positive (TP), True Negative (TN), False Positive (FP), and False Negative (FN) for each class. Based on TP, TN, FP, and FN, the evaluation metrics of Acc, F1, B-Acc, and B-F1 can be formulated as follows:

1. **Accuracy:** Accuracy calculates the recall of all samples:
  5 $$\,{\rm{Acc}}\,=\frac{{\sum }_{c=1}^{C}T{P}_{c}}{{\sum }_{c=1}^{C}(T{P}_{c}+F{N}_{c})},$$
  where *C* denotes the total class number, *TP*_*c*_ denotes the True Positive of class *c*.
2. **F1 score:** The overall F1 score is the arithmetic mean of the F1 scores for all classes:
  6 $$\begin{array}{c}{{\rm{Precision}}}_{c}=\frac{T{P}_{c}}{T{P}_{c}+F{P}_{c}},\,{{\rm{Recall}}}_{c}=\frac{T{P}_{c}}{T{P}_{c}+F{N}_{c}},\\ {{\rm{F1}}}_{c}=\frac{2{{\rm{Precision}}}_{c}\cdot {{\rm{Recall}}}_{c}}{{{\rm{Precision}}}_{c}+{{\rm{Recall}}}_{c}},\,\,{\rm{F1\; score}}=\frac{1}{C}\mathop{\sum }\limits_{c=1}^{C}{{\rm{F1}}}_{c}.\end{array}$$
3. **Balanced Accuracy:** Balanced accuracy calculates the average recall rate for each class:
  7 $$\,{\rm{B}} \mbox{-} {\rm{Acc}}=\frac{1}{C}\mathop{\sum }\limits_{c=1}^{C}\,{{\rm{Recall}}}_{c}.$$
4. **Balanced F1 score:** Balanced F1 score is the weighted average of F1 scores for each class, with the weight being the proportion of samples in each class to the total sample size.
  8 $${w}_{c}=\frac{{N}_{i}}{N},\,\,{\rm{B}} \mbox{-} {\rm{F1}}=\mathop{\sum }\limits_{c=1}^{C}{w}_{c}{{\rm{F1}}}_{c},$$
  where *N*_*c*_ denotes the samples number of class *c*, *N* denotes the total samples size.

### Image classification accuracy with advanced data augmentation

As shown in Table 2, we evaluate the proposed dataset over various classification models with regular training and advanced data augmentation. Our proposed HybridMix augmentation consistently improves the baseline training with an average improvement of 1.57% across eleven models, including 1.42% for mobile networks, 1.01% for CNNs, and 2.25% for ViTs. The results indicate that HybridMix can enhance the generalization capability for TCMP recognition. The models with various sizes often present distinct performances. The lightweight MobileNetV3, ShuffleNetV2, and EfficientNet-B0 models are often deployed over resource-limited mobile devices, achieving 83% ~ 86% accuracy. The accuracy is generally improved as the parameter size increases. The best models for CNN and ViT are ConvNeXt-Tiny and Swin-Base, which achieve 89.43% and 89.64%, respectively. The results demonstrate that large models reach the best accuracy performance. Given the class imbalance in the dataset, we also report balanced accuracy (B-Acc), F1 score, balanced F1 score (B-F1) alongside overall accuracy. We can observe that HybridMix leads to consistent improvements on B-Acc, F1, and B-F1 metrics. The results show that our models trained by HybirdMix work well on the class imbalance scenario.

**Table 2:** Image classification accuracy (%) over multiple models with baseline training and advanced HybridMix augmentation on our proposed dataset.

Type	Model	Parameters	Baseline				HybridMix			
			Acc	F1	B-Acc	B-F1	Acc	F1	B-Acc	B-F1
Mobile	MobileNetV3-Large^36^	4.6M	81.76	80.19	80.02	81.69	82.99	81.54	81.29	82.88
	ShuffleNetV2^37^	2.8M	82.13	80.59	80.50	82.03	83.65	82.21	81.99	83.59
	EfficientNet-B0^38^	4.4M	84.62	83.16	83.10	84.53	86.13	84.87	84.68	86.07
CNN	ResNet-50^33^	24.1M	86.51	85.23	85.13	86.45	87.47	86.24	86.08	87.41
	DenseNet-161^34^	27.1M	87.76	86.58	86.43	87.68	88.78	87.66	87.50	88.72
	ConNeXt-Tiny^39^	28.1M	88.18	87.03	86.96	88.09	89.43	88.32	88.20	89.35
	RegNet-X^35^	14.6M	87.75	86.46	86.40	87.66	88.57	87.37	87.30	88.52
ViT	Swin-Tiny^25^	27.8M	**88.27**	**87.20**	**87.13**	**88.22**	89.39	88.23	88.15	89.31
	Swin-Small^25^	49.1M	86.96	85.66	85.54	86.88	89.13	87.99	87.91	89.10
	Swin-Base^25^	87.1M	87.88	86.76	86.65	87.83	**89.64**	**88.55**	**88.49**	**89.63**
	ViT-Base-16^40^	86.0M	83.25	81.75	81.66	83.15	87.19	85.92	85.74	87.08

The **bold** numbers denote the best results.

### Image classification accuracy with various input resolutions

As shown in Table 3, we investigate the effectiveness of the proposed dataset with various input resolutions. We conduct experiments over multiple classification models to increase the confidence of the results. We can observe that the accuracies of classification models generally improve as the input resolution increases. When the input resolution is increased from 224 × 224 to 336 × 336, the classification models achieve an average accuracy gain of 0.84%. When the input resolution is increased to 448 × 448, the average accuracy improvement is up to 1.65%, and RegNet-X achieves the best 89.54% accuracy. Moreover, we also observe that the larger input resolution enhances B-Acc, F1, and B-F1 metrics consistently. The experimental results demonstrate that classification accuracy benefits from larger input resolutions.

**Table 3:** Image classification accuracy (%) over multiple models with various input resolutions on our proposed dataset.

Model	Input Resolution											
	224 × 224				336 × 336				448 × 448			
	Acc	F1	B-Acc	B-F1	Acc	F1	B-Acc	B-F1	Acc	F1	B-Acc	B-F1
MobileNetV3-Large^36^	81.76	80.19	80.02	81.69	83.24	81.78	81.66	83.16	84.80	83.46	83.35	84.77
ShuffleNetV2^37^	82.13	80.59	80.50	82.03	84.07	82.60	82.46	83.96	85.40	84.00	83.86	85.27
EfficientNet-B0^38^	84.62	83.16	83.10	84.53	85.89	84.55	84.44	85.79	86.97	85.71	85.65	86.89
ResNet-50^33^	86.51	85.23	85.13	86.45	87.59	86.31	86.22	87.51	88.92	87.81	87.70	88.88
DenseNet-161^34^	87.76	86.58	86.43	87.68	87.88	86.74	86.58	87.81	87.95	86.93	87.06	87.82
ConNeXt-Tiny^39^	**88.18**	**87.03**	**86.96**	**88.09**	87.17	85.92	85.81	87.08	86.69	85.38	85.31	86.59
RegNet-X^35^	87.75	86.46	86.40	87.66	**88.78**	**87.66**	**87.59**	**88.72**	**89.54**	**88.84**	**88.74**	**89.87**

### Analysis of accuracy curve during the training process

**Fig. 8:**
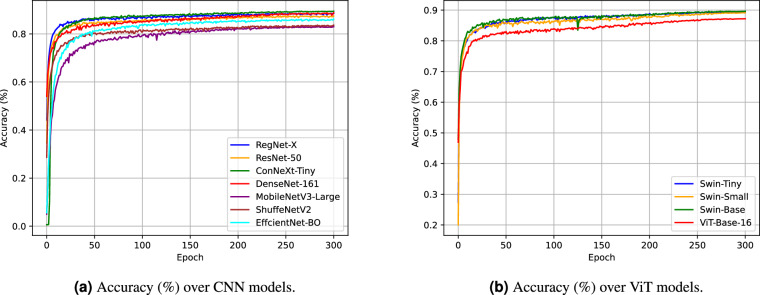
Accuracy of image classification dataset on our proposed dataset.

Figure 8 shows the accuracy curves of the CNN and ViT models during the training process. We find that the accuracy curve often steadily increases as the training epoch proceeds. Accuracy improves rapidly during the first 50 training epochs and tends to grow slowly after the 50th epoch. The network converges at the 300-th epoch and reaches the best accuracy.

### Visualization of learned feature spaces

**Fig. 9:**
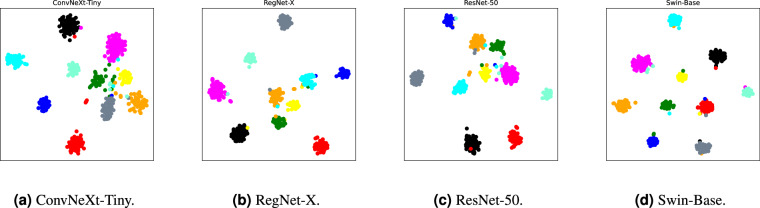
Visualization of learned feature space from random samples by t-SNE^44^.

**Fig. 10:**
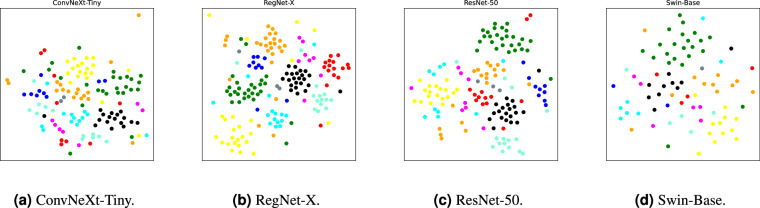
Visualization of learned feature space from hard cases by t-SNE^44^.

Figures 9 and 10 show t-SNE^44^ visualization of learned feature spaces over various networks from random and hard samples, respectively. For clear visualization, we randomly sample 10 classes from the dataset. In figures, each cluster including points with the same color represents a unique class. As shown in Fig. 9, we can observe that each network exhibits well intra-class compactness and inter-class separability. The visualization results indicate that each network learns a discriminative feature space, leading to good classification performance. Moreover, we also analyze the feature space of hard samples in Fig. 10. Here, the hard sample is defined as the misclassified sample by the AI model. We find that the feature space of hard samples is less discriminative than that of random samples. This indicates why hard cases are easy to be misclassified.

### Visualization of attention heatmaps

**Fig. 11:**
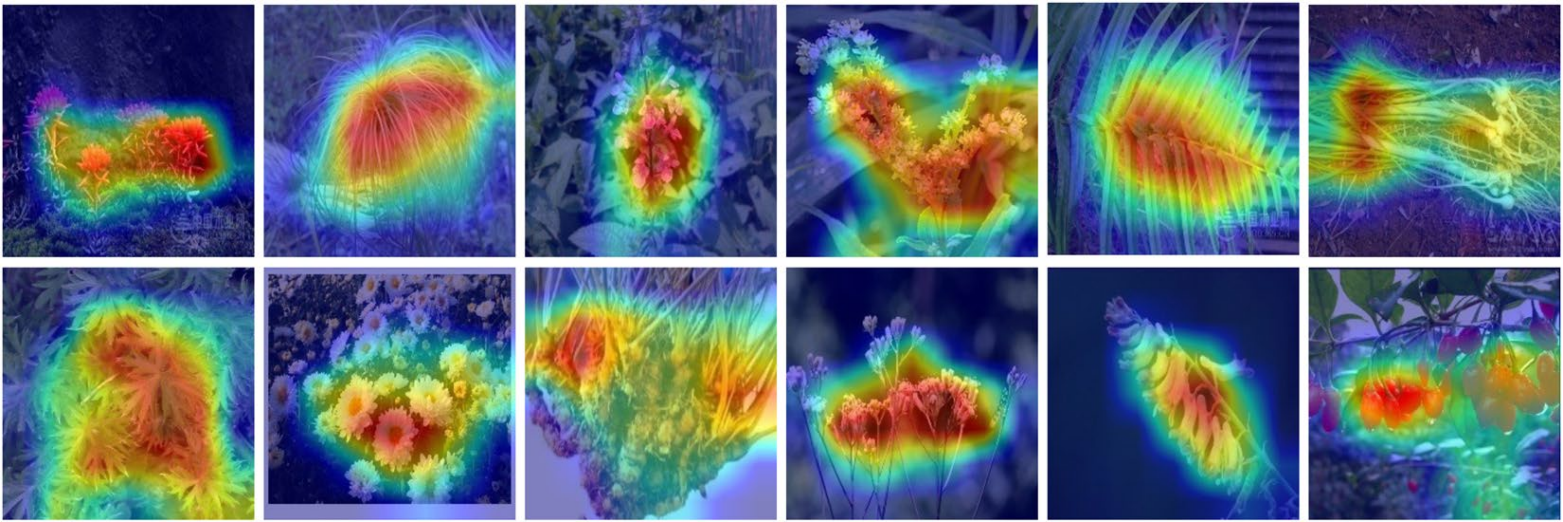
Visualization of attention heatmaps with accurate TCMP-related features by Grad-CAM^45^.

**Fig. 12:**
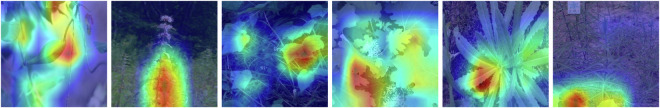
Visualization of attention heatmaps with inaccurate TCMP-related features by Grad-CAM^45^.

Figures 11 and 12 show attention heatmaps using a pretrained ResNet-50 model by Grad-CAM^45^. As shown in Fig. 11, the visualization results indicate that the model trained by our TCMP-300^24^ dataset can exactly capture fine-grained discriminative features for critical parts, such as blossom, stem, leaf, root, fruit, and plant, while ignoring the noise parts. The captured critical features are classification evidence for robust and accurate TCMP recognition. Moreover, we also found a small number of failure cases when the model focuses on inaccurate TCMP-related features, as shown in Fig. 12. This means that AI models may also have potential biases on few TCMP images.

## Usage Notes

**Fig. 13:**
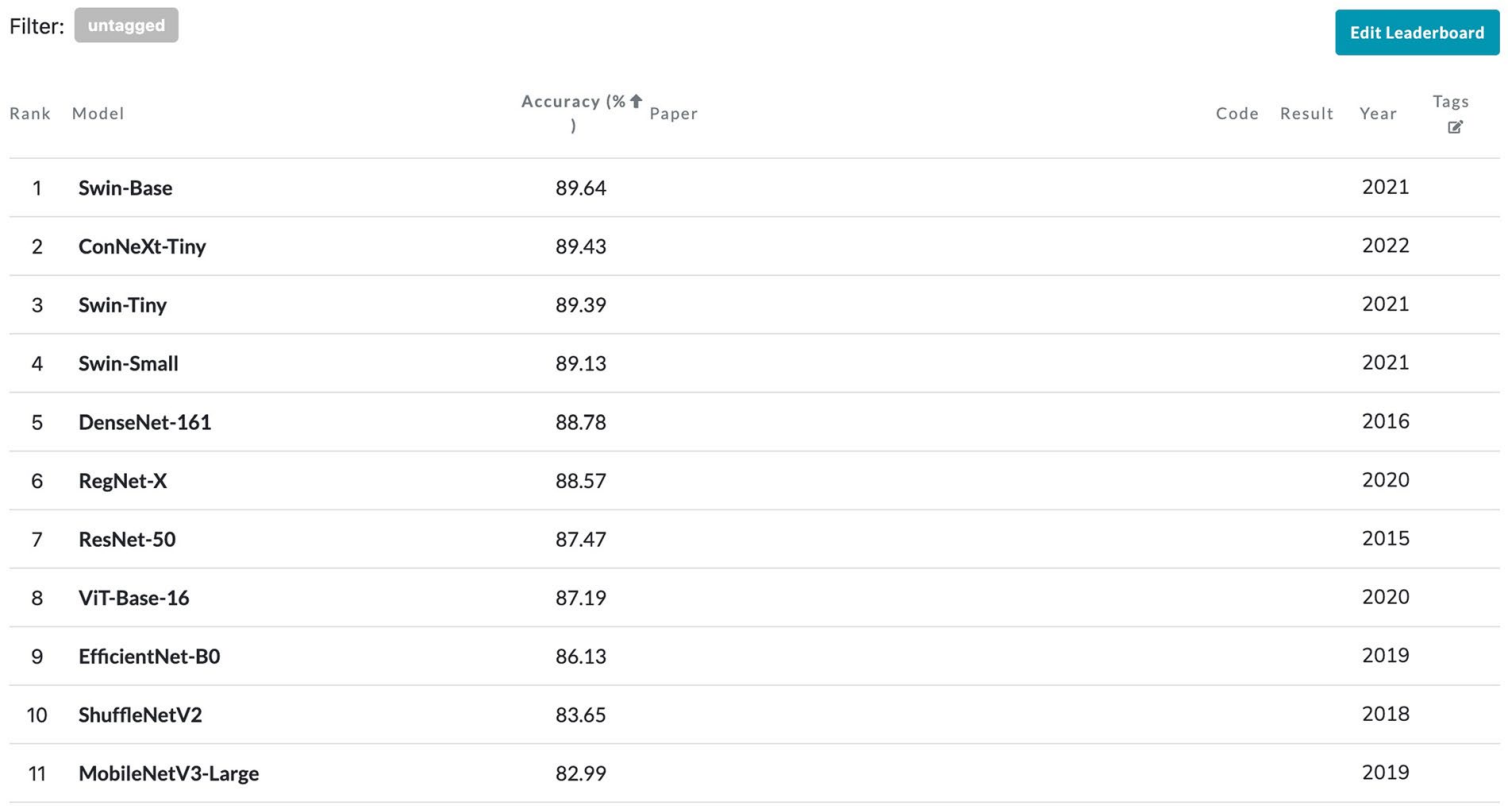
Overview of the online leaderboard for TCMP recognition.

Anyone can access TCMP-300^24^ dataset from figshare platform. The standard PyTorch dataloader module could be called to load the dataset for training and validation. We also release some superior AI models pretrained on the TCMP-300^24^ dataset as baselines and references for future research to explore more powerful AI models. As shown in Fig. 13, we also open a leaderboard of our dataset over paperwithcode at https://paperswithcode.com/sota/image-classification-on-tcmp-300. Anyone can update the best accuracy online to promote the progress of TCMP recognition.

**Fig. 14:**
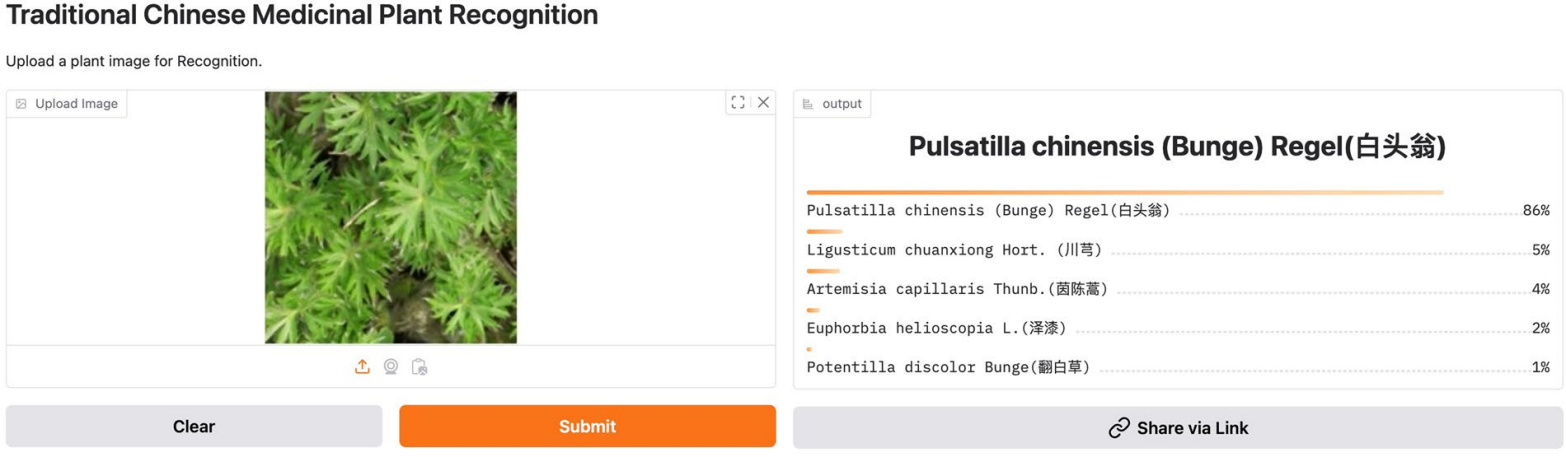
Overview of the online web application for TCMP recognition.

To facilitate clinical practice, we deploy our model into an online web application over HuggingFace. As shown in Fig. 14, anyone can visit this system at https://winycg-tcmprecognition.hf.space/?__theme=system&deep_link=trNDfB9dwx8and upload an image for online plant recognition. Moreover, the system can also output top-5 class probabilities to interpret classification evidence.

Our dataset provides a platform to expand the number of categories and their numbers of images. We release ready-made tools for image crawling and data cleaning. The users can further construct personalized datasets according to their requirements. We hope our TCMP-300^24^ dataset can become a larger-scale dataset with more diverse categories and samples.

## Data Availability

The GitHub repository for the TCMP-300^24^ dataset is available at https://github.com/winycg/TCMP-300. This repository offers detailed tools and guidelines for users to combine the TCMP-300^24^ dataset into their research. It contains comprehensive tools to promote the study with the TCMP-300^24^ dataset and enable customization of workflows for image crawling, data cleaning, data preprocessing, model training, model inference, and model deployment. Moreover, we also provide data analysis tools to plot training curves, visualize t-SNE spaces and Grad-CAM heatmaps.
